# Blood pressure levels and prognosis of intracranial trauma patients with cognitive dysfunction

**DOI:** 10.12669/pjms.304.4930

**Published:** 2014

**Authors:** Weiyu Wang, Junbiao Fang, Bing Lei

**Affiliations:** 1 Weiyu Wang, Department of Neurosurgery, Zhejiang Provincial People’s Hospital, Hangzhou 310014, China.; 2 Junbiao Fang, Department of Anesthesiology, Zhejiang Provincial People’s Hospital, Hangzhou 310014, China.; 3 Bing Lei, Department of Neurosurgery, Zhejiang Provincial People’s Hospital, Hangzhou 310014, China.

**Keywords:** Blood pressure, Cognitive dysfunction, Intracranial trauma, Risk factor

## Abstract

***Objective:*** To evaluate the effects of blood pressure levels on prognosis of intracranial trauma patients with cognitive dysfunction.

***Methods:*** One hundred and twenty intracranial trauma patients enrolled in our hospital from February 2011 to July 2013 were selected, including 40 hypertension and 80 non-hypertension cases. They were investigated by MiniMental State Examination (MMSE) and Montreal Cognitive Assessment (MoCA) scales, and the clinical data were retrospectively analyzed.

***Results:*** Compared with the control group, the MoCA, visuospatial executive function, attention, language, delayed recall, MMSE, orientation and memory scores of the hypertension group were significantly lower. Unconditional Logistic regression analysis showed that age, history of cerebrovascular disease and triglyceride level were the independent risk factors of cognitive function.

***Conclusion:*** The blood pressure levels of intracranial trauma patients were associated with cognitive function, with age, history of cerebrovascular disease and triglyceride level as the independent risk factors. Therefore, it is necessary to control blood pressure level to improve prognosis.

## INTRODUCTION

Intracranial trauma, as one of the critical diseases upon clinical emergency, may give rise to fatal brain herniation.^1^ Generally, intracranial trauma results from accidental fall from aerial work platforms, car accident, heavy collision, and violent fight, etc. Meanwhile, hypertension has become an essential risk factor endangering the health of the elderly and even the youth with economic development as well as changes of lifestyle and dietary habit.^2^ Cognitive function, which is one of the advanced features of the cerebral cortex, is usually damaged encountering some common diseases.^3^^,^^4^ As a crucial psychological process of human, cognitive activity is indispensible for environmental adaptation. It is well known that hypertension affects cerebral cognitive function subtly without leading to severe outcomes. Therefore, cognitive dysfunction is often unperceivable for patients. However, the prognosis of intracranial trauma patients can be significantly improved by cognitive function training during recovery.

Besides affecting brain atrophy significantly, hypertension also influences regulation of cognitive function and dementia eventually.^5^^-^^7^ High blood pressure level accelerates the aggravation of mild cognitive dysfunction, and leads to cardiovascular and cerebrovascular insufficiency and devastating diseases finally by inducing vascular lesions such as intimal hyperplasia, vascular spasm and endothelial dysfunction.^8^ Considering that intracranial trauma is commonly accompanied by cognitive dysfunction without the influencing factors clarified hitherto,^9^^,^^10^ we herein studied the effects of blood pressure levels on the prognosis of such patients.

## METHODS

***Subjects*** *: *Randomized controlled trial and non-repetitive sampling were used in this study, with the sampling equation n ≈ (Confidence interval)^2^ × C^2^/h^2^. One hundred and twenty intracranial trauma patients enrolled in our hospital from February 2011 to July 2013 were selected. *Inclusion criteria:* In accordance with the diagnosis criteria for intracranial trauma; 18-70 years old; with written consent. *Exclusion**** criteria***: Autoimmune disease, hyperthyroidism, severe anemia, and bleeding disorders; severe heart, liver and kidney dysfunctions; mental disorders; failure to complete scales.

 The patients were divided into a hypertension group (n=40) and a non-hypertension group (n=80) according to the diagnosis standards in "2005 Chinese guidelines for the management of hypertension". The gender, age, education background, histories of cerebrovascular disease, smoking and drinking, as well as body mass index (BMI) of the two groups did not differ significantly (Table-I).

***Collection of Clinical Data: ***The patients were investigated by MiniMental State Examination (MMSE) and Montreal Cognitive Assessment (MoCA) scales. The age, gender, education background, histories of cerebrovascular disease, smoking and drinking, drug administration, and blood pressure level were recorded. The levels of cholesterol, triglyceride, high density lipoprotein-cholesterol, low density lipoprotein-cholesterol, fasting blood sugar and 24-hour dynamic blood pressure were examined in laboratory.

***Evaluation on Cognitive Function and Mental Status:***

***MMSE:*** This scale is one of the most influential tools for screening cognitive impairment, aiming to examine orientation, memory, attention, calculation, recall and language abilities. The method is highly reliable and valid with the scores of 0-30. A total score <24 indicates cognitive dysfunction.

***MoCA:*** This scale examines visuospatial executive function, naming, memory, attention, language, abstraction, delayed recall and orientation abilities. The method is highly reliable and valid with the scores of 0-30.

***Statistical Analysis: ***The data were analyzed by SPSS 13.0. The scores were expressed as (mean ± standard deviation) and analyzed by t-test for two independent samples. The numeric data were compared by Chi-square analysis and rank sum test. Multivariate analysis was performed by stepwise multiple regression. P<0.05 was considered statistically significant.

## RESULTS

***Comparison between MoCA Scores: ***The MoCA, visuospatial executive function, attention, language and delayed recall scores of the hypertension group were significantly lower than those of the control group (P<0.05) (Table-II).

***Comparison between MMSE Scores: ***MMSE, orientation and memory scores of the hypertension group significantly exceeded those of the control group (P<0.05) (Table-III).

***Risk Factors of Cognitive Dysfunction: ***The incidence rate of cognitive dysfunction in the hypertension group was 60.0% (24/40) because the scores were lower than 24. Based on the results of unconditional Logistic regression analysis, age, history of cerebrovascular disease and triglyceride level were the independent risk factors for cognitive function (P<0.05) (Table-IV).

**Fig.1:**
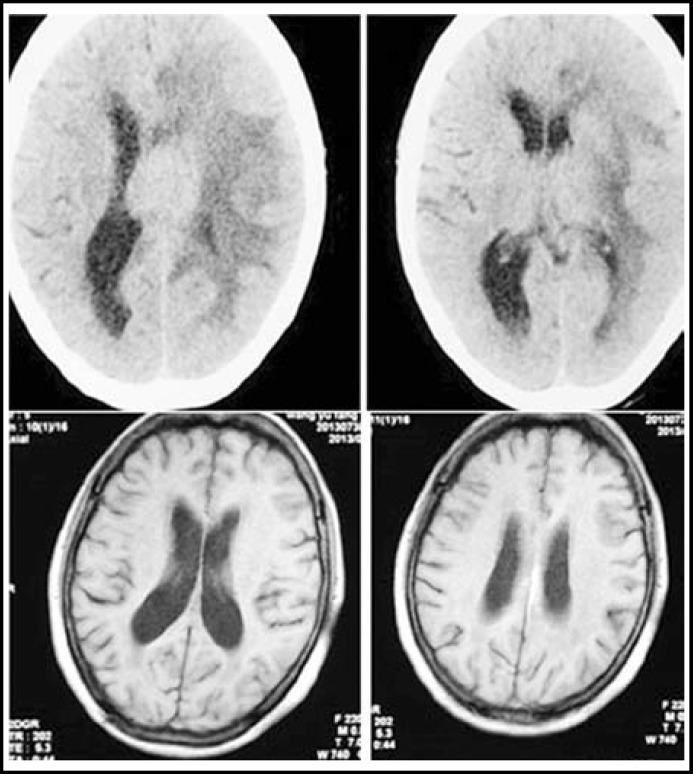
CT examination results before and after clearance of epidural hematoma

***Case Analysis: ***A 33-year-old male, who was in coma after hitting a fence when riding a motorcycle, was rushed to our hospital one hour after the accident. CT examination disclosed considerable epidural hematoma in the left frontoparietal region and midline shift. Emergency operation had cleared the hematoma and decompressive craniectomy had been performed. Postoperative CT examination disclosed that the hematoma was cleared and the midline position was reset (Fig.1).

## DISCUSSION

Hypertension is an important risk factor for cerebrovascular disease, and the most important one for coronary artery disease and cerebral apoplexy. In the last two decades, the awareness, treatment and control rates of hypertension have been raised. However, approximately 40 million patients did not receive effective treatment.^11^ Hypertension results in atheromatous plaque and artery stenosis by damaging endothelial cells, by thickening tunica intima and tunica media, and by accumulating lipids.^12^

(top: before; bottom: after).

**Table-I:** Basic information of the selected patients

***Index***	***Hypertension (n=40)***	***Non-hypertension (n=80)***	***X*** ^2^ *** or t***	***p value***
Gender (male/female)	24/16	50/30	0.412	0.243
Age (years old)	56.32±2.12	55.99±2.47	0.385	0.298
Education background (year)	10.56±1.56	11.12±0.89	0.400	0.255
History of cerebrovascular disease (n)	16 (40.0%)	32 (40.0%)	0.000	1.000
History of smoking (n)	20 (50.0%)	42 (52.5%)	0.128	0.532
History of drinking (n)	15 (37.5%)	30 (37.5%)	0.000	1.000
BMI ((kg/m^2^)	22.95±0.36	23.44±0.39	0.498	0.213

**Table-II:** Comparison between MoCA scores (x±s).

***Score***	***Hypertension (n=40)***	***Non-hypertension (n=80)***	***t***	***p value***
Total	24.11±2.85	26.41±0.76	9.455	0.000
Visuospatial executive function	4.01±0.18	4.41±0.13	6.782	0.000
Naming	2.62±0.46	2.68±0.47	0.423	0.276
Memory	7.66±0.36	7.96±0.61	1.587	0.062
Attention	5.71±0.18	6.00±0.20	11.251	0.000
Language	1.90±0.81	2.55±0.84	12.288	0.000
Abstraction	1.23±0.41	1.33±0.41	0.869	0.098
Delayed recall	2.65±0.45	3.42±0.18	10.781	0.000
Orientation	5.92±0.42	5.98±0.38	1.236	0.071

**Table-III:** Comparison between MMSE scores (x±s).

***Score***	***Hypertension (n=40)***	***Non-hypertension (n=80)***	***t***	***p value***
Total	24.35±1.25	27.80±1.40	11.625	0.000
Orientation	8.88±0.67	9.96±0.28	13.524	0.000
Memory	1.92±0.32	2.45±0.24	10.254	0.000
Attention and calculation	3.76±0.50	4.90±0.30	1.201	0.078
Recall	0.94±0.04	0.98±0.05	0.485	0.312
Language	9.52±0.45	9.45±0.58	0.622	0.211

**Table-IV:** Multivariate analysis of cognitive function

***Variable***	***β***	***p value***	***OR (95%CI)***
Age	0.052	0.023	1.056 (1.033-1.085)
History of cerebrovascular disease	0.651	0.002	1.785 (1.385-1.952)
Triglyceride level	0.852	0.042	1.289 (1.265-1.722)

Intracranial trauma not only jeopardizes cognitive function, but also renders the prognosis unsatisfactory.^13^ Moreover, long-term hypertension ultimately attenuates cognitive function by triggering neuronal degeneration and cell death due to cerebral arteriosclerosis and decreased cerebral blood flow.^14^ Particularly, in case hypertensive intracerebral hemorrhage accounts for 90% of the overall cerebral hemorrhage, cognitive dysfunction is induced posterior to cerebral infarction and cerebral atrophy. History of cerebrovascular disease is the independent risk factor for cognitive impairment.^15^^,^^16^ In the meantime, hypertension patients may have suffered from subclinical morphological changes before intracranial trauma, including asymptomatic cerebral infarction, vulnerable zone (e.g. white matter) hypoperfusion induced by chronic ischemia of deep perforating arteries, together with subcortical (e.g. memory, cognitive speed), executive and control dysfunctions.^17^

It is generally believed that hypertension evidently affects the some aspects of cognitive function such as calculation, patterning, judgment, summarization and attention. In contrast, the functions closely associated with normal human life, such as knowledge, memory, orientation, thinking and language, remain basically intact. Meanwhile, MoCA and MMSE scales have high sensitivity and detection rate for the patients with mild cognitive dysfunction, and they have become the most widely acceptable and facile scales. In this study, the total MoCA score, visuospatial executive function, attention, language and delayed recall scores of the hypertension group were significantly lower than those of the control group (P<0.05). Total MMSE score, orientation and memory scores of the hypertension group were significantly higher than those of the control group (P<0.05).

The incidence of cognitive dysfunction in the hypertension group was 60.0%, as indicated by the scores below 24. According to the results of unconditional Logistic regression analysis, age, history of cerebrovascular disease and triglyceride level were the independent risk factors for cognitive function (P<0.05). Since age is a crucial risk factor, the patients older than 60 years were required to receive cognitive function examinations regularly. High blood lipid level is prone to inducing cognitive function disorders by undermining cerebral artery and capillary endothelial cells, accelerating the development of atherosclerosis, and decreasing cerebral blood flow. Furthermore, cerebrovascular disease-induced hypertension is generally concomitant with reduced cerebral blood flow and cortical perfusion, as well as interactions between degenerative lesion and vascular damages, which may be responsible for the low MMSE scores.^18^

Of the 40 hypertension patients, 21 cases were treated with calcium antagonists + diuretics, 4 cases were given converting enzyme inhibitors + β receptor antagonists, 4 cases were treated with calcium antagonists + angiotensin antagonists + diuretics, and 11 cases were prescribed angiotensin antagonists +β receptor antagonists + diuretics. Their blood pressure levels were effectively controlled, but the prognosis should be further observed.

In summary, the blood pressure levels of intracranial trauma patients were associated with cognitive function, with age, history of cerebrovascular disease and triglyceride level as the independent risk factors. Therefore, it is necessary to control the blood pressure level to improve prognosis.
